# Innovations in Quail Welfare: Integrating Environmental Enrichment, Nutrition and Genetic Advances for Improved Health and Productivity

**DOI:** 10.1002/vms3.70424

**Published:** 2025-06-12

**Authors:** O. E. Oke, K. M. Oliyide, O. A. Akosile, A. I. Oni, E. O. Adekunle, B. O. Oyebanji, O. P. Aremu, I. M. Adeoba, T. A. Eletu, J. O. Daramola

**Affiliations:** ^1^ Department of Animal Physiology Federal University of Agriculture Abeokuta Nigeria; ^2^ Centre of Excellence in Poultry Sciences University of Lome Lome Togo; ^3^ Department of Animal Sciences Obafemi Awolowo University Ile‐Ife Nigeria; ^4^ Department of Mechanical, Bioresources and Biomedical Engineering University of South Africa Pretoria South Africa

**Keywords:** artificial intelligence, enrichment, health, productivity, quails, welfare

## Abstract

The demand for ethical and sustainable poultry production is driving up the importance of quail welfare. Because quail meat and eggs are in high demand, quails are frequently kept in harsh production environments that may harm their health and well‐being. This review examines contemporary developments that combine enhanced nutrition, environmental enrichment and genetic improvements to improve quail well‐being. By concentrating on these essential elements, we offer a thorough synopsis of tactics that can improve the welfare and productivity of quails in industrial environments. Enhancing the environment for quail has shown to be a successful strategy. It has been demonstrated that adding naturalistic elements, such as perches, materials for dust bathing and different lighting, reduces stress, promotes natural behaviours and enhances general health. We analysed the influence of innovative feeding on immunity, gastrointestinal health and stress resilience, emphasising the importance of personalised nutritional interventions in welfare improvement. Additionally, there are encouraging prospects to improve quail welfare thanks to genetic developments. Results are starting to emerge from selective breeding initiatives designed to increase stress tolerance, disease resistance and environmental adaptation. The potential of genetics and biotechnology in developing quail strains that are resilient to welfare challenges and highly productive is covered in this review. This review highlights the potential of integrating environmental enrichment, precision nutrition and genetic innovations to enhance quail welfare in intensive systems. These science‐based strategies improve bird well‐being and productivity, responding to growing consumer demand for ethically produced animal products. Their adoption can support sustainable farming, increase profitability and promote a more responsible and resilient poultry industry.

## Graphical abstract

This review highlights integrated strategies, environmental enrichment, precision nutrition and genetic advancements for enhancing quail welfare in intensive systems. Naturalistic housing, targeted feeding and selective breeding improve health, stress resilience and productivity.

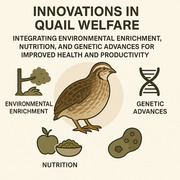

## 1 Introduction

Poultry welfare has emerged as a pivotal concern in modern animal production systems, closely tied to the ethical, economic and sustainability pillars of livestock agriculture. Although poultry species are often praised for their efficiency in converting feed into high‐quality protein, the intensification of production systems has raised significant challenges related to the physical and psychological well‐being of birds (Kpomasse et al. 2021; Akosile et al. 2023; Kpomasse et al. 2023; Oni et al. 2024; Wilcox et al. 2023). Addressing welfare issues requires a multidimensional approach that integrates environmental, nutritional and genetic strategies. Yet, in practice, these factors are often managed in isolation, resulting in suboptimal outcomes that compromise bird health, productivity and consumer trust. The environment in which birds are raised can have a significant impact on their well‐being (Wilcox et al. 2023). Early bird welfare begins with providing optimal hatchability conditions for newly hatched chicks (Fares et al. 2023). By providing a suitable environment, proper nutrition and selective breeding, poultry producers can help to ensure the health and well‐being of their birds (Fushai et al. 2025). This not only benefits the animals themselves but also contributes to the sustainability and success of the poultry industry (Dalmau et al. 2024). As the demand for poultry products continues to grow, it is important that producers prioritise the welfare of their birds to maintain a thriving and ethical industry.

Chickens are known as the primary source of eggs among poultry species; however, as Japanese quails grow and mature quickly, many countries are developing Japanese quail production as a sustainable source of egg and meat production, and it is gaining popularity all over the world (Laurence et al. 2014). According to the Netherlands Ministry of Agriculture, Nature, and Food Quality (2021), the quail population is the second largest among poultry. Katerynych and Pankova (2020) indicated that the yearly rise in quail meat consumption of 5–10% indicates that quail meat has prospective market potential and its production is increasing in more countries like India, Bangladesh, Pakistan, Australia and Ukraine. On the basis of the data that is available, it can be inferred that over 80% of quail eggs are produced in tropical and subtropical regions, specifically India, China, Japan, Indonesia, Mexico and Brazil (Shahbandeh 2021).

Japanese quails are known for their early onset of egg production, usually beginning at about 40 days of age. Under proper management, a female quail can produce more than 250 eggs per production cycle (Al‐Shammari et al. 2019; Lukanov and Pavlova 2020). For optimal fertility, breeding flocks are typically organised in a ratio of one male to four females (Lukanov and Pavlova 2020). However, despite their prolific laying capacity, domesticated Japanese quails have lost the natural brooding instinct of their wild ancestors. This limitation means that natural incubation and chick‐rearing are uncommon, requiring producers to rely on artificial incubators to ensure successful hatching and survival. The Japanese quail (*Coturnix japonica*) is increasingly recognised as an ideal species for small‐scale poultry production and scientific research due to its rapid growth, early sexual maturity, efficient feed conversion and small body size. These birds can be slaughtered as early as 5 weeks of age and begin laying eggs around 42 days, making them suitable for beginner farmers and low‐input production systems (Chelmonska et al. 2008; Kirrella et al. 2023). Their docile nature and ease of handling also contribute to their suitability for urban backyard farming and ornamental purposes (Lukanov et al. 2018). In addition to their commercial value, quails are widely used as model organisms in research because of their short generation interval, low maintenance requirements and relevance in biomedical studies, including allergy research, embryological development and vaccine production (Huss et al. 2008; Antonelli et al. 2024). Furthermore, their meat and eggs—rich in protein, essential micronutrients and low cholesterol—are gaining popularity in the functional food industry (Antonelli et al. 2024). The growing global demand for quail products and their expanding use across research and production systems highlight the importance of addressing their welfare needs (Kiani 2022; ELSaidy et al. 2021; Nasr et al. 2016).

Despite these advantages, the intensification of quail production has raised significant concerns about the birds’ welfare. Although quails are resilient, their welfare can be compromised at various stages of production, ranging from hatchery management to housing transitions, beak trimming and environmental stressors such as heat (Hedlund et al. 2022; Janczak and Riber 2015). The cumulative impact of these stressors affects their productivity and manifests in behavioural and physiological disruptions. For example, prolonged exposure to suboptimal temperatures has been shown to reduce egg production, impair feed intake and trigger metabolic and oxidative stress responses (Del Vesco et al. 2020; Oke et al. 2021; 2024).

Thermal stress is especially detrimental during the laying phase when optimal thermoneutral conditions (22–24°C and 58%–62% RH) are crucial for maintaining egg quantity and quality (Castro et al. 2017). Deviations from these parameters can result in lighter eggs, reduced laying rates and impaired reproductive function (El‐Tarabany 2016; Wasti et al. 2020). These challenges are exacerbated by the confined and often barren environments in which quails are housed—conditions that restrict natural behaviours such as dust bathing, foraging and exploration, leading to frustration and abnormal behaviours (Wasti et al. 2020).

Improving quail welfare in modern production systems requires a multifaceted approach that combines environmental, nutritional and genetic innovations. Environmental enrichment is critical in allowing quails to express natural behaviours, thereby reducing stress and abnormal behaviours such as feather pecking and pacing. Nutritional strategies, such as optimising amino acid balance, incorporating functional feed additives (e.g., antioxidants, probiotics and phytogenics) and tailoring diets to different life stages, can mitigate physiological stress, enhance gut health and improve immune competence. Additionally, advances in genetic selection and breeding, particularly the development of thermotolerant and docile strains, offer long‐term solutions for enhancing resilience and productivity without compromising welfare. This review argues that improving quail welfare requires a holistic approach integrating three key innovations: environmental enrichment, nutritional optimisation and genetic advancements. Environmental enrichment provides opportunities for behavioural expression and stress reduction; nutritional strategies can mitigate physiological stress and enhance resilience; and genetic tools offer the potential for selecting traits linked to both productivity and welfare. By synthesising recent findings across these domains, this article aims to provide a forward‐looking framework for advancing welfare standards in quail production systems.

## 2 Global Perspectives on Quail Welfare

**FIGURE 1:**
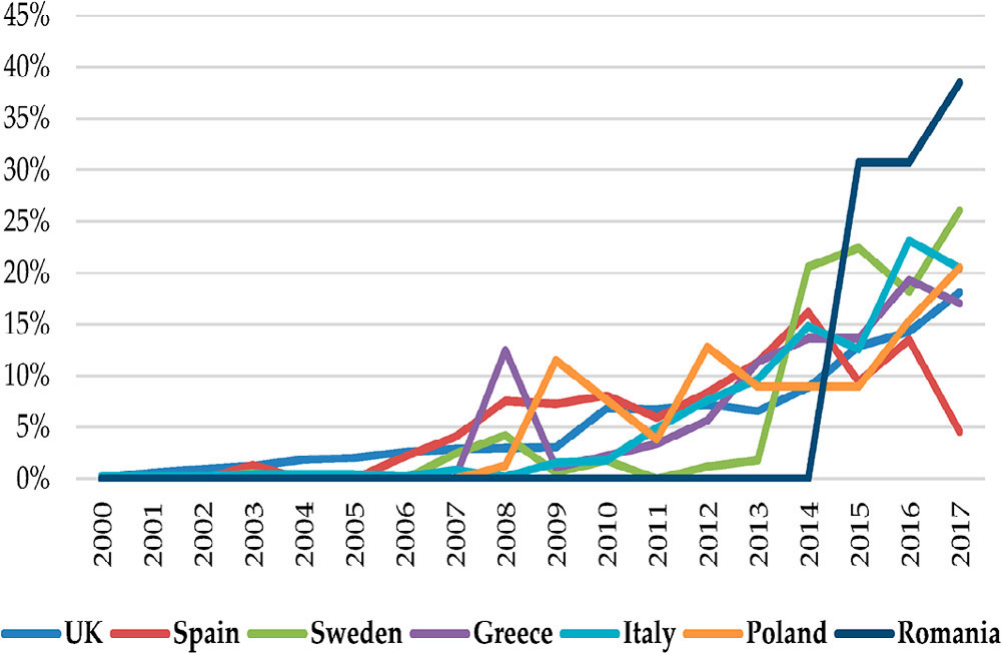
Percentage of annual increase of new meat products with animal welfare claims in several European Union (EU) countries (Pejman et al. 2019).

Quail welfare standards exhibit considerable variation across different regions, influenced by cultural practices, economic conditions and regulatory frameworks. Numerous studies have demonstrated that attitudes towards animal welfare differ significantly across countries and cultures. These attitudes are often correlated with economic development and the extent of modernisation in animal farming practices within a given country (European Commission 2005). Notably, consumers in southern European countries display a greater propensity to pay a premium for products that adhere to more stringent animal welfare standards compared to their counterparts in northern European countries and the United Kingdom (Veissier et al. 2008). Furthermore, consumers in Sweden and the United Kingdom express greater trust in animal production systems that uphold animal welfare standards in conjunction with interventions from public institutions (Veissier et al. 2008). Figure 1 shows the percentage of annual increase of new meat products with animal welfare claims in several European Union (EU) countries. This highlights how animal welfare attitudes can vary from culture to culture and country to country.

Globally, nutrition and environmental enrichment innovations have been increasingly adopted to enhance quail welfare, particularly in commercial production systems. In Europe, countries, like France and Italy, have advanced the integration of precision nutrition tailored to the genetic lines of Japanese quail, focusing on improving gut health, enhancing disease resistance and increasing stress tolerance (Whitehead 2002; Minvielle 2004). In Asia, especially in Japan and China, nutrigenomics‐based dietary strategies are being developed to align with local breeds’ physiological needs, improving productivity and welfare outcomes.

Similarly, environmental enrichment practices are being implemented in various regions. For instance, in Turkey and Brazil, the inclusion of perches, dust baths and visual barriers in quail housing has been shown to reduce stress and promote natural behaviours such as foraging and dust bathing (Taskin and Karadavut 2017). In Scandinavian countries, welfare legislation supports the routine use of enrichments to meet behavioural needs in line with higher animal welfare standards. These strategies improve the psychological well‐being of quails and lead to better production performance, demonstrating a practical balance between welfare and productivity on a global scale.

In North America, animal welfare standards are shaped by a combination of governmental regulations and industry‐driven initiatives. The US Department of Agriculture (USDA) plays an important role in enforcing federal legislation related to the transportation and slaughter of livestock. Additionally, the USDA provides leadership in collaborative programs and allocates funding for research focused on animal well‐being (Reynnells 2004). In Canada, the Canadian Food Inspection Agency (CFIA) establishes minimum animal welfare requirements through its legislative framework (Doonan et al. 2009). Industry‐led initiatives, including voluntary standards and auditing programs developed by retail organizations and commodity groups, are increasingly influential in determining welfare practices for poultry and livestock (Mench 2003; Thaxton et al. 2016). These standards encompass various facets of animal care, including housing, handling and slaughter methods (Oke et al. 2015). Nonetheless, challenges persist in areas such as the growth rates of broilers, housing systems for layers and humane slaughter practices.

In Europe, quail welfare is addressed through legislative measures such as the EU's Council Directive 1999/74/EC, which sets forth minimum standards for the protection of laying hens, including quails (European Food Safety Authority [EFSA] Panel on Animal Health and Animal Welfare 2023). This directive encompasses regulations on housing systems, stocking densities and enrichment provisions, all aimed at enhancing the overall well‐being of quails within commercial production environments.

In the Asian context, quail farming is a prevalent practice, yet welfare standards exhibit considerable variability across different countries (Arya et al. 2018). This variability arises from the coexistence of traditional farming techniques alongside modern, intensive production systems, which leads to diverse welfare considerations. Efforts to standardise welfare practices across Asian nations are currently in progress, with organisations such as the World Organisation for Animal Health (WOAH) (OIE) providing guidelines aimed at enhancing quail welfare throughout the region.

In Africa, quail welfare is shaped by a blend of traditional farming methods and nascent commercial production systems. The continent faces unique challenges in animal welfare due to these traditional practices, emerging commercial systems and constrained resources (Abbas 2014; Masiga and Munyua 2005). Historically, free‐range systems were prevalent; however, the transition towards intensive production has raised significant concerns regarding animal welfare, particularly within large‐scale operations (Masiga and Munyua 2005). In South Africa, quail farming is increasingly recognised as a viable protein source, yet farmers encounter obstacles such as high feed costs, disease prevalence and restricted market access (Mnisi et al. 2021). Cultural norms, socioeconomic factors and inadequate dissemination of information impede the adoption of international welfare standards within African communities (Njisane et al. 2020). Nonetheless, initiatives focusing on knowledge transfer and capacity building are emerging to promote awareness of best welfare practices (Abbas 2014). Enhancing animal welfare through sustainable methodologies and community empowerment holds the potential to improve productivity, elevate product quality and contribute to food security across Africa (Njisane et al. 2020).

A comparative analysis of quail welfare standards across various regions reveals both commonalities and disparities (Dalmau et al. 2024). Universally, aspects such as access to clean water, appropriate nutrition and disease prevention are critical to quail welfare; however, specific requirements and implementation strategies may differ based on regional contexts. Global challenges in maintaining high welfare standards for quails include reconciling traditional practices with modern advancements, addressing resource limitations in certain areas and fostering compliance with welfare regulations (Dalmau et al. 2024). Collaborative efforts involving government entities, industry stakeholders and animal welfare organisations are vital for effecting positive change and elevating quail welfare standards worldwide. By gaining a comprehensive understanding of the diverse approaches to quail welfare across regions, stakeholders can work towards establishing best practices that prioritise the well‐being of quails while considering the unique circumstances and challenges faced in each locale. This holistic approach is essential for promoting sustainable and ethical quail production practices on a global scale.

Cultural and economic determinants substantially influence quail welfare practices. In numerous cultural contexts, quail are regarded as a vital source of nutrition and revenue, which subsequently affects their treatment and care. In certain societies, quail are reared in traditional backyard environments, where economic limitations often result in minimal attention to their welfare (Kinyua 2022). Such conditions can lead to issues like poor nutrition and insufficient healthcare, adversely affecting the birds’ welfare. Economic considerations further impact quail welfare practices. In areas where quail farming constitutes a major income source, there may be an impetus to enhance production levels, potentially at the expense of animal welfare. This can lead to intensive farming methodologies that emphasise production volume over the quality of life for the quail. Additionally, cultural perceptions of animals can significantly influence quail welfare practices. In some cultures, there may be a lack of awareness or concern regarding the welfare of quail, resulting in practices that do not support their well‐being.

The welfare of quail is significantly influenced by a range of international policies and regulations designed to ensure their humane treatment and care. In the United States, the Animal Welfare Act (AWA) establishes standards for the care and treatment of animals, including quail, particularly within research environments. This legislation, enforced by the USDA's Animal and Plant Health Inspection Service, has undergone multiple amendments since its initial enactment in 1966 (Cardon et al. 2012). The AWA encompasses most warm‐blooded animals, with certain exceptions, and mandates that facilities housing quail provide adequate housing, nutrition and veterinary care to ensure their well‐being (Mulcahy 2017).

In the EU, comprehensive animal welfare legislation, including directives for farm animals and poultry, governs the welfare of quail (Gavinelli and Lakestani 2010; Blokhuis 2004). The Treaty of Lisbon, enacted in 2009, recognised animals as sentient beings and elevated animal protection to a standalone article (Gavinelli and Lakestani 2010). EU regulations address various aspects of animal welfare, such as housing, transport and slaughter, with the European Commission playing a critical role in implementing and enforcing these regulations through robust control systems (Dwinger and Lambooij 2012). Although EU legislation has positively impacted animal welfare and influenced global practices, there remains a need for a general animal welfare law and species‐specific regulations, as many animal species are still unprotected (Broom 2020). The EU's animal welfare strategy includes the Animal Welfare Platform and integrates scientific information from organisations like the EFSA into policy formulation (Broom 2020). Consumer concerns and sustainability issues continue to drive animal welfare policies within the EU (Blokhuis 2004). Specifically for quail, the Council Directive 2007/43/EC establishes minimum standards for the protection of chickens kept for meat production, which can extend to similar poultry species like quail. This directive underscores the importance of appropriate housing conditions, feeding and health monitoring. Additionally, the European Commission is revising animal welfare legislation to enhance protections for all farmed animals, including quail, with a focus on traceability and combating illegal trafficking.

Globally, the WOAH sets international standards for animal health and welfare, including guidelines applicable to quail. These guidelines advocate for best practices in animal husbandry and welfare, ensuring that quail are raised in environments that meet their physical and behavioural needs (WOAH 2023). Moreover, the EFSA has undertaken comprehensive evaluations concerning the welfare of ducks, geese and quail, offering scientific guidance to inform policy decisions and enhance welfare standards throughout the EU (EFSA 2023). The continuous engagement among various stakeholders, including governmental bodies, non‐governmental organisations and the agricultural sector, is crucial for progressing quail welfare regulations on a global scale. This ensures that such regulations not only adhere to existing legal frameworks but also address evolving welfare concerns.

## 3 Regional Differences in Quail Production and Welfare Practices

Differences in animal welfare standards worldwide appear to be influenced by income, culture and religion (Zewdie et al. 2020; Sinclair et al. 2022). As income levels rise, the consumption of livestock products increases, initially with greater demand for quantity, followed by higher quality standards and a preference for premium meat and other animal products (Weis 2013). Animal welfare regulations often include aspects of providing a public good (McInery 2004). In countries with more advanced development stages, governments are typically more effective at delivering these sophisticated public goods. In the EU, poultry welfare receives more legislative focus than in many other regions (Leone 2020). This focus is partly due to specific characteristics of the production environment. Additionally, policymakers argue that EU consumers are increasingly concerned about the welfare of production animals (European Commission 2005).

The development of quail farming for commercial purposes has progressed unevenly across the globe. In certain regions, such as Japan, there is a notable emphasis on high egg production, whereas countries like Spain and France focus significantly on meat production. In contrast, the Netherlands, Germany and the United Kingdom have little to no commercial quail production (Minvielle 2004). Quail production and welfare practices exhibit considerable variation across regions, shaped by local environmental conditions, cultural practices and economic factors (Batool et al. 2023). In tropical and arid regions, heat stress emerges as a critical challenge to the welfare and productivity of quail (Alagawany et al. 2017). Research indicates that elevated ambient temperatures can negatively impact growth performance, reproductive success and overall health in quail populations, necessitating specific management strategies to alleviate these effects (Bhawa et al. 2023). In contrast, temperate regions place a greater emphasis on optimising housing conditions and nutrition to enhance the welfare of quail (Kiani 2022). For instance, ensuring sufficient space and environmental enrichment can markedly improve the behavioural welfare of quail by reducing stress and promoting natural behaviours (Laurence et al. 2015). Additionally, the incorporation of technological advancements in poultry farming, such as automated feeding and climate control systems, is increasingly prevalent in developed regions, contributing to improved welfare outcomes (Ramankevich et al. 2022).

Cultural perspectives on animal welfare significantly influence production practices (Estévez‐Moreno et al. 2021; Garcia and McGlone 2022). In certain regions like Europe, there is an increasing recognition and promotion of humane treatment for quail, which has led to implementing welfare‐friendly practices (Pejman 2022). These practices include providing enriched environments and preventing overcrowding, which is crucial for ensuring quail's physical and psychological well‐being (Nordi et al. 2012). Recognising these regional variations is essential for the development of effective welfare strategies that address the specific needs of quail populations in diverse contexts. Collaborative efforts among researchers, farmers and policymakers are imperative to advance best practices and enhance the welfare of quail on a global scale.

## 4 Regulatory Framework and Guidelines on Quail Welfare

The regulation of animal welfare, including housing and management conditions, is guided by various international and national frameworks. The WOAH (formerly OIE) provides global standards for the welfare of farmed animals, including recommendations on housing, handling and behavioural needs, which are applicable to avian species, although specific references to quails are limited (WOAH 2023).

At the regional level, the EU enforces general welfare requirements through Directive 98/58/EC concerning the protection of animals kept for farming purposes. This directive emphasises the importance of allowing species‐specific behaviours and proper housing for all farmed animals, including birds (European Council 1998). Although there is no specific directive for quails, welfare recommendations are increasingly being extended to minor poultry species under national schemes (EFSA 2010).

In North America, particularly the United States, quails are not covered under the Humane Methods of Slaughter Act, and federal welfare regulations often do not explicitly address their housing conditions. However, voluntary certification programs, such as Certified Humane, Animal Welfare Approved and Global Animal Partnership (GAP), have begun including standards for non‐conventional poultry species, focusing on enriched environments, adequate space and behavioural opportunities (Humane Farm Animal Care 2020; GAP 2023).

In Africa, welfare legislation is still developing, but the African Union Inter‐African Bureau for Animal Resources (AU‐IBAR) has acknowledged the need to harmonise animal welfare practices and integrate them into livestock production systems (AU‐IBAR 2019). Quails are rarely covered under specific laws, but advocacy and policy dialogues are promoting the inclusion of welfare considerations for all species. Overall, although legislation specifically targeting quail welfare remains sparse, international guidelines and evolving certification schemes provide a framework for improving their welfare. Research evidence supporting the benefits of environmental enrichment is critical for informing these standards and encouraging broader adoption.

## 5 Common Welfare Challenges in Quail Farming

### 5.1 Behavioural Restriction and Environmental Enrichment

Confined environments in intensive quail production systems limit the birds’ ability to express natural exploratory behaviours, often resulting in stress, boredom and the development of abnormal behaviours such as feather pecking and pacing. These are major welfare concerns. Laurence et al. (2015) demonstrated that using environmental enrichments, such as perches, significantly increased behavioural diversity and reduced stress indicators in confined quails. Similarly, Taskin and Karadavut (2017) found that incorporating sand baths and visual barriers improved comfort, reduced aggressive behaviours and enhanced growth performance. These findings highlight the value of environmental enrichment strategies in promoting welfare in confined settings.

### 5.2 Housing Systems: Cage vs. Floor Pen Housing

Inadequate housing, particularly battery cages, is a significant welfare concern in quail farming. Battery cages severely restrict movement, preventing quails from engaging in essential behaviours such as foraging, dust bathing and social interaction. As a result, quails housed in such systems often exhibit stereotypic behaviours indicative of chronic stress. In contrast, floor pen systems—especially those enriched with forages, perches and hiding spots—provide more natural behaviour expression opportunities. Studies by El‐Sheikh (2016) and Ramankevich et al. (2022) support the view that floor pen housing improves welfare and productivity due to the broader range of social and environmental stimuli available.

### 5.3 Stocking Density

Stocking density is another critical factor affecting welfare in quail farming. High stocking densities can increase resource competition and stress and reduce reproductive performance. El‐Sheikh (2016) reported that maintaining a lower stocking density of about 20 birds/m^2^ significantly enhanced productivity and reproductive success compared to birds raised under higher density conditions.

### 5.4 Thermal Stress and Environmental Conditions

Environmental conditions, particularly temperature extremes, also present substantial welfare challenges. Quails are most comfortable within a thermoneutral zone of 18–24°C. Exposure to temperatures above this range leads to physiological stress, reduced feed intake, impaired feed efficiency and diminished productivity (Alagawany et al. 2017). One promising strategy for mitigating heat stress involves the development of thermotolerant quail lines through genetic selection and crossbreeding, thereby enhancing resilience to high ambient temperatures (Abou‐Elkhair et al. 2020; Dosoky et al. 2021).

### 5.5 Comparative Performance in Different Rearing Systems

Research comparing cage and floor‐rearing systems for Japanese quails has yielded mixed results. Some studies indicate that cage systems promote better body weight gain, feed intake, feed conversion ratio and survivability and also offer improved hygiene (Razee et al. 2016; Azam et al. 2021). On the other hand, floor systems have shown higher feed intake and body weight in certain studies (Azam et al. 2021). Economically, cage systems demonstrated slightly higher, though statistically insignificant, net profit and return on investment. Interestingly, both systems showed no significant differences in carcass traits, immune responses or oxidative stress parameters (Razee et al. 2016; Azam et al. 2021). These mixed outcomes suggest that system selection should consider not only productivity but also the behavioural and welfare needs of the birds.

## 6 Environmental Enrichment

The provision of environmental enrichment is crucial in improving the welfare of quails, and it is particularly essential in addressing their behavioural and physiological needs. Environmental enrichment is a critical component of housing and management strategies aimed at improving the welfare of quail in intensive production systems. Like other poultry species, quails exhibit a range of natural behaviours such as foraging, social interaction and nesting, which are essential for their well‐being. When these behaviours are restricted due to inadequate housing or environmental conditions, it can lead to stress and various welfare issues (Laurence et al. 2015; Ramankevich et al. 2022).

The effect of environmental enrichment on the physiology of quail is also of notable importance, and this has been reported in terms of the growth rate, egg production and overall health of the species. Wengerska et al. (2022) reported that quails raised in enriched environments showed improved production and better egg quality compared to those raised in standard housing conditions. Quails' environment can be enriched physically and socially. Enrichment interventions, such as providing pecking objects, varied substrates, perches and dust‐bathing areas, encourage the expression of natural behaviours, reduce stress‐related responses and ultimately contribute to better health and productivity outcomes.

Laurence et al. (2015) and Wengerska et al. (2022) outlined that enrichments, such as nest boxes, scratching surfaces and sand baths, contributed progressively to the laying rate, egg weight and body weight of the quails. Furthermore, a comparative study by Roshdy et al. (2010) on floor pen housing and battery cages showed that laying rate, hatchability and egg quality were better in the floor pen housing compared to the battery cages, whereas mortality was also significantly lower in the floor pen house. Similarly, housing that allows for natural light and space can improve the quality of eggs by enhancing their nutrient composition, yolk colour and shell strength (Alig et al. 2023; Erek and Matur 2024). According to research, quail stress levels caused by crowded or inadequately ventilated housing can have a detrimental effect on internal egg quality metrics, including albumen height and Haugh unit, as well as egg production rates and shell thickness (El‐Tarabany 2016a, 2016b).

Additionally, the quality of poultry products is significantly influenced by housing systems, as these environments directly affect bird behaviour, physiology and welfare. For example, because of their increased physical activity, birds in enriched or floor‐based systems frequently have superior muscle development and meat tenderness than those in traditional cages (Marchewka et al. 2023; Sun et al. 2023; Kaya et al. 2024). Studies on Japanese quails found that enriched environments improved immune response, lowered stress levels as indicated by reduced corticosterone levels and reduced stress‐induced immunosuppression (Nazar and Marin 2011; Nordi et al. 2012; Ramankevich et al. 2022; Wengerska et al. 2022). The importance of environmental enrichment has also been reported in the latency of comb temperature recovery after exposure to stress (Ross et al. 2020).

### 6.1 Types of Environmental Enrichment

#### 6.1.1 Physical Enrichment

Physical enrichments are a common category of enrichments usually provided in quail housing to aid environmental cues and, consequently, their welfare. Structurally, nests, perches, sand bath, scratching surfaces, tunnels and hiding spots have been provided in their houses and have been reported to significantly benefit the birds, notably in the reduction of stress, facilitation of natural behaviour, reduce stereotypic pacing and improvement of overall welfare (Laurence et al. 2015; Taskin and Karadavut 2017).

Perches are one such example and are commonly provided in quail housing. Perches are important to quails, providing them the opportunity to exhibit their natural behaviour, such as perching, which is necessary to alleviate the physical and psychological impact of confinement in battery cages and promote brain and musculoskeletal development (Pohle and Cheng 2009). Research has shown that the availability of perches significantly impacts the behaviours exhibited by laying hens, improving their spatial skills (Gunnarsson et al. 2000; Habinski et al. 2017). Additionally, the availability of perches from an early age has been found to promote the development of perching behaviour in chicks, helping maintain their behavioural repertoire as they grow (Heikkila et al. 2006). Moreover, Charles (2024) showed that various forms of enrichment, including physical enrichment, could significantly improve animal well‐being and productivity. However, great caution must be taken when reporting the effects of enrichments as they are often complex and dependent on factors such as the novelty of the enrichment and duration of exposure to such enrichment items. Fairhurst et al. (2011) pointed out that short‐term enrichment may initially act as a stressor, whereas long‐term enrichment can lead to acclimation. To limit this inconsistency, evidence‐based enrichment strategies have been encouraged to ensure the treatment of research animals based on ethical guidelines to ensure higher reliability of scientific research (Charles 2024).

**FIGURE 2:**
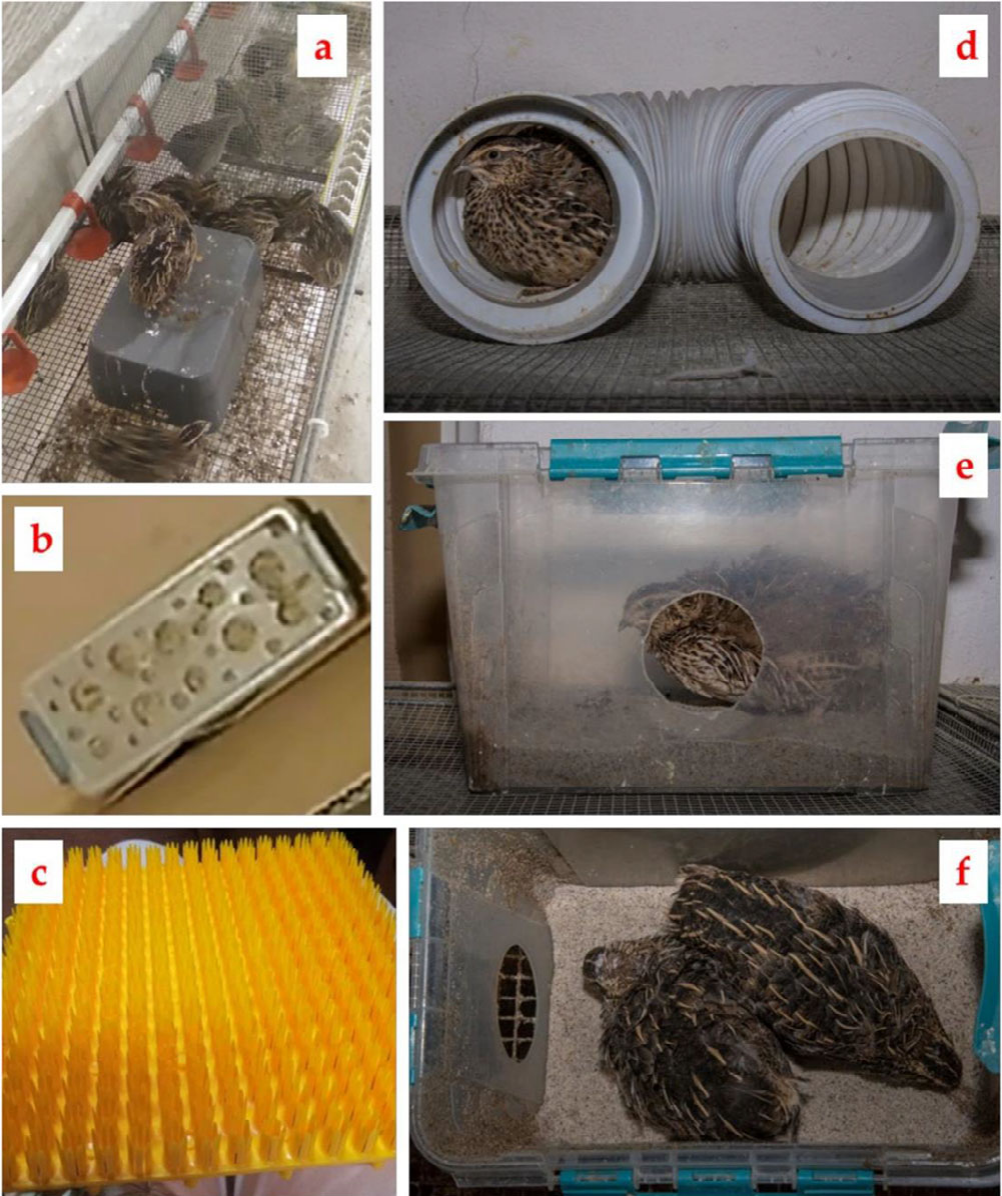
Some enrichments applied into the Japanese quail cages (a) nest box, (b) feeder, (c) scratching surface, (d) tunnel, (e, f) sand box (Ramankevich et al.,2022).

Sand bathing is another enrichment needed for quails. Studies on caged egg‐laying hens have demonstrated that providing them with access to dust bathing improved their welfare (Barnett et al. 2009). Similar reports have also been documented on Japanese quails. If quails have access to sand baths, they have been observed to spend as much as 25% of their daily activities (Miller and Mench 2005). A sandbox could be added as an enrichment to give the birds sand baths, but it also serves as a source of mineral elements and improves the strength and quality of the eggshells (Ipema, Bokkers et al. 2020). Furthermore, foraging enrichment is one type of environmental enrichment that can reduce stereotypical behaviours, boost leg health and increase bird activity (Ipema, Bokkers et al. 2020, Ipema, Gerrits et al. 2020). As shown in Figure 2, several enrichments could be applied into the Japanese quail cages, nest box, feeder, scratching surface, tunnel and sand box (Ramankevich et al. 2022).

Nest boxes are another critical form of physical enrichment commonly offered to laying hens, allowing them to express their natural nesting behaviours and improving their productivity with the potential to reduce stress (Cronin and Hemsworth 2012). Naturally, *C. japonica* will make their nests in less crowded areas containing rough and scattered plant materials, a behaviour commonly seen during incubation and clutch formation. Nest boxes containing dark grey vertical stripes are preferred by Japanese quails (Sarda‐Palomera et al. 2012). Nest boxes provide safety for eggs and help reduce the stress associated with egg‐laying and nesting behaviours. The availability of suitable nesting sites has been shown to improve the welfare of hens by allowing them to express their natural nesting instincts, leading to better egg production and lower incidences of egg‐related stress (Hemsworth and Edward 2020).

#### 6.1.2 Sensory Enrichment

Sensory enrichment refers to all enrichments specifically directed to stimulating the primary sense organs. This involves stimulations through sight, sound, taste, touch and smell to promote the exhibition of their natural behaviours and generally improve their overall welfare (Jones 2001, 2002). Sensory enrichment is essential for enhancing the welfare of animals in captive settings by providing mental stimulation and promoting natural behaviours. Diverse sensory experiences, such as auditory and visual stimuli, influence postnatal development by extending responsiveness to maternal cues and aiding in interpreting environmental signals (Carlsen and Lickliter 1999; Honeycutt and Lickliter 2003). Enrichments utilising sight, touch, taste, hearing and smell uniquely recruit positive affective states (Mulholland et al. 2021). They also encourage the expression of instinctual behaviours like foraging and digging, vital for cognitive development and mental engagement (Sabuncuoğlu et al. 2018; Elbaz et al. 2023). Furthermore, sensory interventions such as lighting modifications and controlled acoustic stimulation significantly reduce stress and improve emotional well‐being, with specific auditory stimuli promoting relaxation and better welfare outcomes (Sabuncuoğlu et al. 2018; Elbaz et al. 2023). By integrating sensory enrichment strategies, caregivers can create dynamic environments that support animals’ mental and physical health.

Light modification has been used frequently to improve welfare, and both light intensity and colour are commonly modified to mimic natural conditions, increase photoperiod and improve animal behaviour, welfare and productivity. Colour is the first enrichment that is important to quail in their environment. Research has shown that birds perceive colours differently from humans and are more sensitive to colours closer to red (Kiani 2022). A scratcher with different colours was most often chosen as the first object of enrichment for quail. Taylor et al. (2003) also examined the impact of perch colour on the selection of nest sites and the rate of response in egg‐laying hens, and they concluded that when perches were coloured differently, the birds responded more quickly and successfully. In action, providing a natural light cycle with dawn and dusk simulations has been reported to help regulate circadian rhythms, thereby leading to healthier animals. The use of auditory stimuli in production houses has recently gained elevated attention, and natural sounds or music are being introduced in production settings to stimulate the auditory senses. Jones (2001) and Jones (2002) reported that auditory stimulation through radio play, combined with tactile enrichment using string devices, enhanced neural development, reduced fear and promoted foraging behaviour. Similarly, Taskin and Karadavut (2017) reported that auditory stimuli improved the welfare of quails raised at different stocking densities. All these reports suggest that auditory stimulation is beneficial to the welfare of quails; however, further research is neededto determine the specific genre of music and the optimal intensity that is beneficial to the birds.

Besides light modification and auditory enrichment, texture modifications that provide tactile stimulation are also used to induce sensory stimulation. Texture modification is provided in various ways, including modified surfaces that animals can explore, such as smooth, rough and varied textures that encourage natural behaviours like scratching, climbing and digging. It has been found that the provision of texture modification can significantly promote physical activities, which is vital for mental and physical health in birds through the enrichment of their sensory experiences (Mulholland et al. 2021). It has also been reported in bobwhite quails that the provision of tactile enrichment significantly affected their sensory development and responsiveness, and augmented prenatal tactile stimulation altered postnatal auditory and visual responsiveness to maternal cues (Carlsen and Lickliter 1999). Incorporating sensory enrichment into quail housing promises to be a welcoming advancement capable of reducing stress by fostering an engaging and dynamic environment for the quail.

#### 6.1.3 Housing and Social Enrichment

Quails are social animals, and group housings are typically preferred for social animals because they facilitate natural social interaction, thus enhancing their overall welfare. Housing has been shown to be one of the most important non‐genetic factors influencing the reproductive, behavioural, healthy and productive traits of hens (Roshdy et al. 2010). In the husbandry of poultry, environmental enrichment or modifications can be used to improve animal welfare, lessen fear and aggression and modify social behaviour since they increase behavioural options and enhance biological functioning (Kiani 2022). Many studies on the social behaviour and environmental enrichment of Japanese quail, a common laboratory and production species, have been conducted. The choice of social proximity in Japanese quail was found to be influenced by social housing as opposed to singleton dwellings by Miller and Mench (2005).

Research shows that quails kept in groups exhibit more natural behaviours, such as foraging and socialisation, than those kept in individual housing. The effect of group housing on productivity and reproductivity has also been reported. Roshdy et al. (2010) and El‐Sheikh (2016) reported that floor pens improved egg production, fertility and hatchability of the birds compared to those reared in battery cages; however, a report by Karousa et al. (2015) found higher egg production in cage‐raised birds than floor‐raised birds. This discrepancy may be due to differences in environmental conditions or breeds of the birds. Individual housing may also be necessary in certain circumstances. For instance, individual housing is considered the best housing option for aggressive as well as sick animals to eliminate the risk of injuring other members of the flock and prevents the spread of diseases (Miller and Mench 2006).

The effects of social enrichment have been observed in the social behaviours and aggression in quails. In quails housed in groups, aggression often arises due to competition for resources such as food, space and mates (Wechsler and Schmid 1998). Moreover, attempts from dominant individuals to assert social dominance over others may prompt others to respond with aggression (Wechsler and Schmid 1998). Oftentimes, this can manifest in various forms, leading to physical injuries from chasing and pecking and sometimes subordination of individuals, limiting their ability to properly benefit from common resources. Research indicates that higher stocking densities can aggravate these aggressive behaviours, making it crucial to provide adequate space to mitigate aggression and promote social harmony (Wechsler and Schmid 1998; Miller and Mench 2006).

The effect of social isolation on aggression has also been reported. Social isolation has been studied and reported to lead to increased incidents of bird‐to‐bird aggression upon introduction or reintroduction into social groups (Ross et al. 2019). Quails previously isolated have also been observed to exhibit a heightened fear response when introduced into existing flocks; this has implications for disruptingsocial dynamics and inducing stress. Conversely, quail housed in enriched environments with adequate social companions tend to display more positive social behaviours and lower aggression, highlighting the importance of social enrichment in their housing. By testing the effects of various hormone treatments, Schlinger and Callard (1990) showed that aggression response is mediated by the aromatisation of testosterone to oestradiol.

### 6.2 Innovations and Best Practices: Case Studies of Successful Enrichment Strategies

Innovations in quail husbandry have increasingly focused on enrichment strategies that enhance the welfare and productivity of these birds. One case study is that reported by Ramankevich et al. (2022). In the study, the author investigated the behavioural and physiological indicators of quails whose housing was enriched with nest boxes, scratchers, plastic tunnel pipes, sandbathing boxes and improved feeder boxes. Their behaviours were then monitored, and blood samples were collected for biochemical analysis. It was therefore reported that the presence of environmental enrichment reduced the incidence of behavioural disturbances in the Japanese quail. In terms of stress, the author found that the individuals kept in enriched cages with greater ability to exhibit their natural behaviours had lower stress levels compared to those kept in unimproved cages (Ramankevich et al. 2022).

Similarly, Nazar and Marin (2011) investigated the impact environmental enrichments have on avian immune responses using Japanese quails and evaluated whether combining them with chronic stressors can help counteract the negative impact of stress on the immune system of the quails. While housing some birds in environmentally enriched boxes and the rest in non‐enriched boxes, half of the birds in each category were subjected to chronic stress for 15 min, and their immune responses were assessed. The outcome of this experiment showed that placing the birds in an enriched environment effectively improved their immune response and mitigated the detrimental effects of exposure to chronic stressors (Nazar and Marin 2011).

### 6.3 Technological Advancements in Environmental Enrichment

Technological innovations have significantly propelled the use of environmental enrichments in quail production, and several instances of these advancements have been reported (Choukidar and Dawande 2017; Kostarev et al. 2022). One of the notable adoptions of technology into the incorporation of environmental enrichment into quail production is the use of automated systems for monitoring and adjusting environmental conditions (Kostarev et al. 2022). These systems have been used to regulate temperature, humidity and daylight hours, creating optimal living conditions for best welfare consequences and optimum productivity. Studies have also shown that these technological advancements do not only enhance the comfortability of the birds but also facilitate the exhibition of natural behaviours, particularly when there are physical enrichments for the birds to interact with (Kostarev et al. 2022).

Another key technological advancement in environmental enrichment is the use of specialised feeding systems that promote foraging. These systems can include movable feeders that change location periodically, thereby stimulating the quails to explore their environment for feed (Miller and Mench 2006). Additionally, incorporating varied substrates and materials for dust bathing and nesting has fulfilled their biological needs, further enhancing their overall well‐being (Scholz et al. 2010; Monckton et al. 2020).

Although automated methods, including cameras, sensors and climate controllers, have significantly increased the accuracy and efficiency of quail production monitoring, they are not perfect. Trained human observation can better identify subtle behavioural or physiological indicators of concern, such as changes in posture, social interactions or early signs of disease, than these technologies. Farmers must thus take a well‐rounded strategy, combining technology with regular visual examinations and practical welfare evaluations. To ensure that staff members can accurately understand automated data and act promptly when technology is unable to provide a complete picture, staff training remains essential. To ensure complete animal care, technology should ultimately be used as a supplement to human attention, rather than as a substitute.

Even though environmental enrichment has many positive welfare effects, like encouraging natural behaviours, lowering stress levels and increasing physical activity, there are certain difficulties in putting it into practice. For example, adding enrichment materials or devices may raise production costs and management complexity, particularly in large‐scale operations (Jacobs et al. 2022). If not properly managed, some enrichment objects could become sources of contamination or harm. Furthermore, not every enrichment technique produces the same results for various quail strains or production methods, and variables like stocking density, housing layout and environmental circumstances might affect how effective an enrichment strategy is (Spieß et al. 2021; Ramankevich et al. 2022). To prevent unforeseen welfare or performance effects, enrichment techniques should be carefully chosen, routinely assessed and designed to meet the demands of particular flocks and production objectives.

## 7 Nutritional Advances and Their Impact on Quail Welfare

Advances in nutrition are essential for improving the welfare of quail because they meet their physiological and behavioural needs. Diets that are balanced and satisfy quail's calorie, protein and micronutrient needs to encourage healthy growth and guard against deficiency that could predispose the birds to stress or diseases (Shastak and Pelletier, 2023). Probiotics, prebiotics and phytogenic substances are functional feed additives that promote intestinal health, strengthen the immune system and lower the risk of infection—all of which minimise the need for antibiotics (Shehata et al. 2022). Additionally, customised feeding techniques, such as precision feeding, guarantee a steady nutrition supply, reducing stress and competition while eating. These dietary advancements improve quail physiological health while lowering aggressiveness and mortality, resulting in a more humane production system.

In 2018, 350 million tonnes of soy were produced; 85% of that amount was intended for animal feeds (Ritchie and Roser 2022), with the remaining portion going directly into human food (Voora et al. 2020). Environmental and socioeconomic issues are posing a growing threat to animal feed's reliance on soybeans as its main source of protein. For the foreseeable future, soybeans will remain a significant commodity, but the industry faces serious sustainability and social issues, such as deforestation, biodiversity loss, greenhouse gas emissions, overuse of herbicides and violations of human rights (Voora et al. 2020). Additionally, the presence of antinutritional factors limits the amount of plant proteins that can be added to aqua and poultry feeds; as a result, insects could present an intriguing substitute (Dawood and Koshio 2020). On the other hand, insects like black soldier fly larvae (BSFL) offer a viable substitute protein supply (Attivi et al. 2019; Mlaga et al. 2020; Attivi et al. 2023). Insects have been explored as a possible poultry feed substitute because of their high nutrient content and low environmental impact (Bovera et al. 2016). Insects offer high‐quality protein with a desirable amino acid profile, can be produced sustainably from organic waste, and require little land and water. Because insects provide advantages for animal welfare, the environment and nutrition, they have gained importance as feed ingredients in chicken farming in recent years (Spartano and Grasso 2021).Several promising characteristics make insects and their products one of the potential substitute feedstuffs to increase the sustainability of the livestock industry. These include short productive cycles, low water requirements (since water is frequently obtained directly from the feeding substrate), limited space requirements for farming and the suitability of some species for mass rearing (Smetana et al. 2021). Insects consume organic waste streams, such as agricultural and industrial byproducts and other feedstuffs deemed waste and lacking commercial value. Theyrecycle these materials into valuable nutrients that can be effectively incorporated into the diets of various animals that produce food, such as fish (Henry et al. 2015), poultry (Shaviklo 2023) and rabbits (Cullere et al. 2022).

### 7.1 Incorporation of Black Soldier Fly (BSF) in Quail Feeding

The different insects fed to quail are shown in Table 1. According to Kawasaki et al. (2019) and Chu et al. (2020), black soldier fly meal (BSFM) is a good source of protein and energy that is rich in vitamins, minerals and saturated, monounsaturated and polyunsaturated fatty acids (PUFA). A wide variety of organic wastes can be bio‐converted by BSF to create nutrient‐rich animal feeds (Surendra et al. 2020). According to studies conducted in a highly productive pilot plant, if BSF is fed to livestock, it may be more advantageous than feeding them soy meal or fishmeal (Smetana et al. 2019).

**TABLE 1:** Insects fed to quail.

Type of insect	Proportion (%)	Effects	Reference
BSF prepupa	5 of inclusion	Increase body weight, feed intake, average daily growth and lower FCR	Elangovan et al. (2021)
*Tenebrio molitor* larvae	5	Improved their performance, egg physicochemical traits and shelf life	Dalle Zotte et al. (2024)
Yellow mealworms	2, 2.5, 4, 5 and 8 of inclusion	Lower FCR	Elahi et al. (2022)
Housefly	4 and 8 of inclusion	Improved growth	Elahi et al. (2022)
*Spodoptera littoralis* larvae meal	50 and 100	Improved body weight and weight gain	Hatab et al. (2020)

Abbreviation: BSF, black soldier fly.

### 7.2 African Cotton Leafworm (*Spodoptera littoralis (S. littoralis)*)

*S. littoralis* meal is a potentially safe, affordable and healthful alternative protein source because of its exceptional ability to transform low‐nutritive waste products into valuable nutrients. Depending on their ability to store fat and protein in their bodies during the larval stage and the substrate they are rearing on, larvae can have a diet rich in nutrients (Makkar et al. 2014). *S. littoralis* meal is recognised to have a high nutritional value and may be a significant source of protein, carbohydrates, fats, vitamins, minerals and so forth, according to Basiouny et al. (2016).

Growing quail chicks fed *S. littoralis* larvae meal instead of meat and bone meal showed improved growth, feed performance parameters, carcass characteristics, haematological and biochemical indices, and lower production costs than the control group. These results suggest that *S. littoralis* larvae could be a promising new feed ingredient for quail (Hatab et al. 2020).

### 7.3 Yellow Mealworm (*Tenebrio molitor* (TM))

It has been suggested that certain insect species, like the yellow mealworm (TM; YM), housefly, superworm, *Bombyx mori* and BSF (*Hermetia illucens*; BSF), could be used in feeds for animals as well as aquafeeds, primarily to replace soy and fish meals (Makkar et al. 2014). When considering alternate and sustainable feed sources for animals that produce food, such as chickens, the TM is without a doubt one of the most fascinating insect species. According to Moruzzo et al. (2021), yellow mealworms can be cultivated on byproducts and agri‐food sidestreams that are occasionally also thought of as waste. The amount of research articles that have been published in the last 10 years evaluating the potential addition of its protein (i.e., meal) or fat fractions to the diet of various animal species—rabbit (Volek et al. 2021), fish (Henry et al. 2015) and poultry and swine (Hong et al. 2020), demonstrates this. According to earlier studies, when 10% of live TM larvae were administered as nutritional enrichment for laying quails, it improved their performance, egg physicochemical traits and shelf life (Dalle Zotte et al. 2024). This is because chitin, an indigestible polysaccharide that makes up an insect's exoskeleton, has immunostimulant properties and antimicrobial (Islam and Yang 2017). It has been reported that the prebiotic effect of chitin helps broiler chickens fed mealworm meal (MWM) to have improved immune response and disease resistance (Bovera et al. 2015). Additionally, chitin in the diet stops *Salmonella*, *Escherichia coli* and *Salmonella enterica* serovar Typhimurium from growing in broiler chickens (Khempaka et al. 2011; Menconi et al. 2014).

### 7.4 Feed Additives

As an alternative to minimising the difficulties faced by birds bred under heat stress, feed additives like phytogenics can be used. The poultry industry can benefit from the use of feed additives in a number of ways, including increased feed utilisation, improved growth and a decrease in the overall cost of producing poultry (El‐Sabrout et al. 2023). Probiotics, prebiotics, enzymes, organic acids, acidifiers, antioxidants and essential oils are just a few examples of well‐known feed additives (Ilias et al. 2023). Furthermore, vitamins and poultry feed additives have significantly enhanced the productivity of the birds (Zhou et al. 2024). According to Salem et al. (2023), both organic and inorganic acids are used as dietary supplements in poultry nutrition.

Probiotics as a feed additive have proven effective in promoting the development of beneficial microflora (Rashid et al. 2022). They are critical factors in maintaining intestinal health and reducing the colonisation of pathogenic microbes. Consequently, this improvement in chicken productivity has been linked to enhanced broiler performance (Nusairat et al. 2022; Bidura et al. 2023). It has been demonstrated that probiotics, which are microbial cell preparations or portions of microbial cells, enhance health. For ages, probiotics, namely, lactic acid bacteria, have been utilised for a range of purposes, including food production (Song et al. 2012). According to Chandrasekaran et al. (2023), beneficial bacteria in the gut can drive out harmful bacteria and preserve a balanced population of bacteria by vying for resources and attachment sites in the intestine.

### 7.5 Enzymes

Ding et al. (2018) discovered that xylooligosaccharides stimulate the growth of lactobacilli and bifidobacteria in the gut, acting as prebiotics. Xylanase improves gastrointestinal health digestion by administering prebiotics for beneficial bacteria (Van Hoeck et al. 2021). Additionally, the findings of Sultana et al. (2024) revealed that giving Japanese quails xylanase enzyme and probiotic supplements improved their growth, digestibility of nutrients and response to infections, decreased the amount of *Salmonella* and *E. coli* in their cecum and increased the number of *Lactobacillus*.

Quail performance, lipid profiles, antioxidants and economic evaluation were all significantly impacted by Labazyme (1000 and 2000 mg/kg). Due to the presence of various probiotic strains and multiple enzymes that help increase feed conversion efficiency and decrease feed intake, Labazyme operates in a different way. This demonstrated how adding exogenous enzymes increases feed efficiency while lowering feed costs and affecting feed intake. Additionally, a correlation between Labazyme and a number of response variables, including economics, egg production, feed intake and feed conversion efficiency, was established. This signified that Labazyme could be added to quail diets as a feed additive at 1000 and 2000 mg/kg (Al‐Mjbel et al. 2022). The growth and economic efficiency are enhanced as a result of the enzymes’ cooperative action in breaking down the various substrates. This increases the amount of nutrients released from the diet. According to Lei et al. (2018), when diets that included raw and processed pigeon pea seed meal (PPSM) were supplemented with an enzyme, it affected the growth and nutritional digestibility of the chicks of Japanese quails.

### 7.6 Functional Oils

The impacts of functional oils have been studied extensively, but there are few reports on thermally stressed laying quails. The intestinal health of the quail could be preserved by adding a functional oil and probiotic blend to heat‐stressed quail feed and obtaining information that supports egg production with wellbeing. The primary active ingredients in the mixture of microencapsulated functional oils consisted of cashew nut liquid (*Anacardium occidentale*), garlic essential oil (*Allium sativum*), pepper oleoresin (*Capsicum* sp.) and copaiba essential oil (*Copaifera officinalis*). The anacardic acids, sesquiterpenns, monoterpenes, diterpenes, capsaicinoids and capsaicin, which have anti‐inflammatory, antimicrobial and anti‐inflammatory properties, increase the thermotolerance of quails to thermal challenge (Bernardes et al. 2014; Araujo et al. 2020).

Functional oil affects nutritional metabolism by increasing the amount of feed thermally challenged birds consume without raising their body temperature. Incorporating dietary 300 g/t of functional oil in Japanese quail resulted in increased feed conversion, enhanced nutrient processing capacity of the liver, and higher yolk content eggs (Barros et al. 2024). This type of nutritional strategy offers an alternative for raising birds in hot climates. A functional oil with high vitamin C content is pepper oleoresin, which has been demonstrated to lessen the harmful impacts of oxidative stress and could assist in keeping quail's homeostasis stable during thermal challenge (Abdelnour et al. 2018). The antioxidant effects of functional oil compounds could result in reduced body temperature in quails under heat stress (Oliveira et al. 2012).

### 7.7 Naringenin

Due to their potentially beneficial effects on health, flavonoids like naringenin have drawn interest from the chicken industry. Additionally, it has demonstrated efficacy in enhancing gut health and has a favourable impact on controlling cholesterol metabolism (Yang et al. 2022). According to Reda et al. (2024), naringenin supplements may help quails grow better by reducing the number of microbes in their growing gut, increasing body weight and gain and improving FCR blood haematological parameters and antioxidant status. Raising the naringenin dose to 1.5 g/kg diet had the best effects, after which the responses decreased. To prevent any negative effects on quail growth and productivity, naringenin added to feed should be supplemented at the proper dose.

### 7.8 Lycopene

Sahin et al. (2007) experiment showed that lycopene‐supplemented quails could yield lycopene‐rich eggs. Additionally, consuming the eggs may enhance an individual's antioxidant status as indicated by higher serum lycopene and lower serum MDA concentrations. It is feasible to deliver antioxidant nutrients to prevent certain human diseases through the production of functional foods.

### 7.9 Canola Meal (CM)

CM usefulness as a source of protein in poultry diets seems to be constrained by its lower energy content, lower available protein content, and comparatively higher fibre content when contrasted with soybean meal (SBM). Given that the AA profiles of the two oil extraction co‐products are relatively similar, CM may be utilised as a substitute for SBM. However, if safety measures are not taken, some antinutritional factors in CM may have a negative impact on quail performance. To increase canola utilisation in quail diets, multi‐enzyme admixtures could be taken into consideration (Mnisi and Mlambo 2018).

### 7.10 Aspergillus niger

*Aspergillus niger* is a fungus that is used extensively in industry because it is essential to the synthesis of citric and succinic acids. It is also an essential component of many biotechnological applications because it can ferment valuable compounds (Upton et al. 2020; Papadaki and Mantzouridou 2024). The use of readily available and reasonably priced agro‐industrial waste materials, such as citric acid made primarily by microbial fermentation with *A. niger*, has been recommended to achieve environmental sustainability (Stefanolo et al. 2024). The growth and well‐being of Japanese quails could be enhanced with the supplementation with *A. niger* filtrate. Seven organic acids were identified from the growth of *A. niger* on agro‐industrial residue of faba bean, including oxalic, ascorbic, maleic, salicylic, lactic, formic and citric. These organic acids reduced intestinal pathogenic bacteria and enhanced intestinal health in the treated birds. They also enhanced antioxidant indices, digestive enzymes, immunity and nutrient digestibility in the quail diet (Al‐Gheffari et al. 2024).

### 7.11 Tryptophan

Due to the inability of the body to synthesise, l‐tryptophan is a nutritionally necessary amino acid for preweaning ruminants and monogastric animals. Specifically, tryptophan (Trp) affects food intake, neurological function and immunity, among other aspects of physiology and nutrition (Yıldırım et al. 2020). Tryptophan also functions as a precursor for melatonin, niacin and serotonin (5‐hydroxytryptamine). Tryptophan has been shown to influence bird behaviour by synthesising important neurotransmitters like serotonin (Yıldırım et al. 2020). The role of the tyrosine kinase pathway in relation to hens’ feather pecking behaviour is also being studied (Birkl et al. 2019). Serotonin is a mediator between the brain and the intestine. The ability of an animal to raise serotonin levels may, therefore, be impacted by increased dietary Trp (Liu et al. 2015). In contrast, the findings of Yildirim et al. (2020) revealed that feeding a high Trp diet is not advised as a strategy to enhance social behaviour, as its action seems to differ from that seen in laying hens. Further studies are needed to elucidate the roles of tryptophan.

### 7.12 Herbal Remedies

Herbal remedies such as valerian, lemon balm and chamomile have been shown to reduce stress and improve the tonic immobility of laying Japanese quail. Japanese quails that were laying showed no change in behaviour when given 500 mg/kg feed containing chamomile, lemon balm and valerian herbs. However, the birds’ tonic immobility time was shortened, with chamomile having the most significant impact (Moraleco et al. 2023). By regulating the alterations in serotonin and norepinephrine turnover in the hippocampus and amygdala, *Valeriana officinalis* root extract reduced both psychological and physical stress reactions in quails (Jung et al. 2014). Moreover, besides keeping the birds sat longer, adding 1.8–5.0 g of chamomile/kg to a Japanese quail's meal decreases aggressive pecking behaviour (Tenório et al. 2017). The tonic immobility period of laying Japanese quails was reduced by the addition of 500 mg/kg feed of chamomile, lemon balm and valerian herbs (Moraleco et al. 2023).

### 7.13 Palm Kernel Cake (PKC)

In the tropics, PKC, a byproduct of deoiling *Elaeis guineensis* nuts, is abundant. PKC's nutritional composition varies, and the technique used to extract the oil affects how much residual oil is present (Ojediran et al. 2020). PKC is considered a feedstuff with moderate amounts of protein, fibre and energy (Sathitkowitchai et al. 2018). PKC is used in animal feeds for pigs (Ojediran et al. 2020), broilers (Alshelmani et al. 2021) and cockerels (Bello et al. 2011). However, little is known about the use of PKC in quail diets, particularly during the laying phase. According to Ojediran et al. (2022), adding 40% PKC to the diets of laying Japanese quail improved the albumen weight, yolk index, shell percentage and cost–benefit ratio, but it also lightened the yolk colour.

### 7.14 Pigeon Pea Seed Meal

According to Akintunde et al. (2018), supplementing quail diets with diets containing both raw and processed PPSM with enzymes can enhance nutrient digestibility and promote optimal growth.

### 7.15 Coconut Cake

According to Moraes et al. (2020), adding 12% coconut cake to Japanese quail diets during the laying phase enhances feed conversion per egg mass, increases the laying rate and increases the percentage of eggshells.

### 7.16 Turmeric Powder

Japanese quail under heat stress benefit greatly from the use of phytogenics; in one study using turmeric powder, feed consumption decreased but had no effect on increases in weight gain and egg production (%), feed conversion or the colour of the yolks of the eggs (Zacaria and Ampode 2021).

### 7.17 Ginger

Yusuf et al. (2015) demonstrated in a different study that the addition of ginger to laying Japanese quail diets, along with probiotics and citric acid, enhanced the quailers’ laying performance, bone characteristics, egg quality, reproductive indexes and feed conversion ratio. Additionally, it has been noted that adding 4 and 8 g/kg of ginger powder to the diet increases weight gain while reducing feed intake (Najafi and Taherpour 2014). According to a study, adding 0.05 g/kg of ginger powder to the diet improved the Japanese quail's egg production, hatching rate, reproductive efficiency and economic output (Abd El‐Galil and Mahmoud 2015). The blood parameters, antioxidant status and productive performance of Japanese quail can be partially enhanced by adding powdered ginger root to their diet. Ginger may also enhance albumen quality as measured in Haugh units and yolk colour. The supplementation of ginger at 0.75 g/kg of powdered was the most effective treatment; the impacts on the performance of quail appeared to be dose‐dependent (El‐kashef and Roshdy 2022).

### 7.18 Clove and *Nigella sativa* (1:1)

The inclusion of dietary *Nigella sativa* and clove in growing Japanese quails enhanced the quail's carcass features, growth performance, liver function and well‐being. These phytogenics demonstrated excellent antioxidant activity. It enhanced the levels of blood glutathione, catalase and superoxide dismutase, as well as the activity of hepatic glutathione. Furthermore, compared to the control group, the MDA concentration in hepatic homogenates of birds receiving CLNS treatment was significantly lower. As a result, using CLNS as a safe growth promoter is recommended. In terms of inclusion level, the diet containing 3.00 mL CLNS/kg had the greatest impact on health and growth performance traits. The efficacy of employing different degrees of cold‐pressed oil mixes comprising 1:1 clove and black cumin oils was explored against the indices of growth and carcass features, as well as blood components of growing Japanese quails (Majrashi 2022). The authors revealed that 3.00 mL CLNS/kg diet had a significant effect on the growth performance and health indicators with respect to the inclusion level.

### 7.19 Historical Overview of the Genetic Advances for Improved Quail Welfare

Quail breeding programs have benefitted from the advancements in the understanding and utilisation of genetic engineering methods through the incorporation of sophisticated techniques in selecting and developing traits of interest (Pang and Zhao 2003; Saxena and Kolluri 2018). The introduction of genetic markers allowed breeders to select specific traits more effectively, leading to improved disease resistance and overall health in quail populations. This shift was significant as it reduced the reliance on antibiotics and other interventions, aligning with modern animal welfare standards.

Recently, the adoption of genomic technologies, such as Clustered Regularly Interspaced Short Palindromic Repeat (CRISPR) and their associated protein (Cas‐9), has revolutionised quail breeding (Ahn et al. 2016). This technology enables more precise gene editing to rapidly introduce traits of interest, such as stress tolerance and improved productivity. This approach not only accelerates the breeding process but also allows for the development of quail that are better suited to various environmental conditions (Lee et al. 2023).

Moreover, breeding programs have been developed to promote behavioural traits because of the broad impact of behaviour on welfare. Genetic selection over the years has been used to establish correlations between behaviour and the physiological status of quails across different conditions. In the open‐field test conducted by Jones et al. (1992), it was shown that the fear level and adrenocortical responsiveness have a positive correlation with quails selected for low‐stress responses, thus showing less fearfulness. Furthermore, Valance et al. (2007) linked fear levels through tonic immobility duration to changes in heart rate variability and autonomic control. Divergent selection for tonic immobility duration and social reinstatement behaviour has resulted in significant and continuous responses over multiple generations, with no evidence of plateauing (Mills and Faure 1991). These studies demonstrate that behavioural traits in quails respond readily to genetic selection, offering the potential to improve welfare and productivity in poultry by enhancing their ability to cope with environmental challenges and social interactions (Jones and Hocking 1999).

### 7.20 Role of Genetics in Welfare and Productivity

Genetic selection has been used to advance productivity and promote welfare in quail husbandry. Research has shown that selective breeding helps address behavioural issues such as fear and feather pecking in poultry (Jones and Hocking 1999). It has also been shown that social reinstatement, emotional reactivity and aggression can be influenced by genetic selection (Recoquillay et al. 2013). Additionally, studies have identified quantitative trait loci (QTLs) associated with behavioural and production traits, revealing potential pleiotropic regions controlling emotional reactivity, body weight, social motivation and egg‐laying onset (Recoquillay et al. 2015). These genetic factors interact with environmental conditions, affecting the development of behaviours such as approach‐avoidance, sexual behaviour and maternal care (Kovach 1974). Understanding the genetic basis of quail behaviour provides insights into animal adaptation to breeding conditions and may have implications for improving animal welfare in poultry production (Recoquillay et al. 2015).

Genetic advancements in Japanese quail have contributed significantly to improved productive performance. For instance, greater mitochondrial nucleotide diversity has been associated with enhanced egg production (Hussein et al. 2020). At the same time, conventional genetic selection for traits such as rapid growth and improved liveability has yielded performance benefits but also raised welfare concerns, including increased susceptibility to metabolic disorders and compromised skeletal health. Recent progress in molecular biology and biotechnology presents new opportunities to address these challenges by enabling the identification and selection of welfare‐related traits. These tools may support the development of genetically optimised birds that balance productivity with enhanced resilience and welfare under commercial production conditions (Aggrey 2010).

### 7.21 Selective Breeding for Welfare Traits

#### 7.21.1 Breeding for Disease Resistance, Stress Tolerance and Behavioural Traits

Genetic advancements in quail welfare have increasingly focused on selective breeding for traits that enhance disease resistance, stress tolerance and positive behavioural characteristics. This approach is crucial for improving the overall health and welfare of quail. Results from breeding programs of quails for stress resistance have yielded promising results, which have since been progressive. However, as demonstrated by the development of high stress (HS) and low stress (LS) quail lines based on corticosterone levels, selective breeding is capable of producing lines with contrasting responses (Satterlee and Johnson 1988). Genomic analysis of these HS and LS lines shows that single nucleotide polymorphism associated with stress‐related pathways supports the genetic basis of stress resistance (Shumaker et al. 2021). It has also been reported that embryo cooling during incubation enhances resistance to stress later in quails’ lives (Rekhletskaya et al. 2023). Besides deliberate selection for stress resistance, selection for some specific traits can have unintended consequences; for instance, Mark and Huston (1973) reported that quails selected for growth on different diets showed reduced tolerance to heat stress compared to no‐selective controls. This result points to the complexity of breeding for specific traits and the need for comprehensive evaluation of multiple factors in selection programs.

Quails have also been bred for behavioural characteristics, and several promising results have been reported by different authors who have explored the subject. Gerken and Peterson (1992) reported low to moderate heritability for social behaviours, dust bathing and fear responses in Japanese quails, whereas Jones and Hocking (1999) reported that welfare could be improved in quails and chickens by selectively breeding to manipulate traits like feather pecking and fearfulness. Recent genome‐wide studies have identified numerous QTLs associated with behavioural traits in Japanese quail. Recoquillay et al. (2015) reported 45 QTLs, 23 of which were related to behavioural traits and 15 specifically linked to social motivation. They also identified pleiotropic regions controlling emotional reactivity and social motivation. Generally, behavioural traits have a significant focus in breeding programs. By selecting a quail that displays less aggressive behaviour and better social interactions, farmers can create a more harmonious environment that reduces stress and injury among birds. This not only enhances the welfare of the quail but also improves overall production efficiency.

### 7.22 Modern Genetic Tools and Techniques

#### 7.22.1 CRISPR and Gene Editing

The application of CRISPR and gene‐editing technologies in enhancing quail welfare presents a promising frontier in animal genetics. CRISPR/Cas9, a revolutionary gene‐editing tool, allows for precise modifications in the quail genome, which can lead to significant improvements in health and overall welfare (Doran et al. 2017).

One way this technology has contributed to the improvement of welfare is through the enhancement of disease resistance (Doran et al. 2017). CRISPR enables genetic engineers to target specific genes associated with immune responses, helping them to develop quails that are more resilient to prevalent diseases., Consequently this reduces the need for antibiotics and other medications that can negatively impact food safety (Doran et al. 2017; Liu et al. 2022). Additionally, gene editing can be utilised to improve growth rate and feed efficiency, which not only benefits farmers economically but also helps reduce the number of birds required to achieve the same goal, thereby reducing overcrowding and competition for resources.

Moreover, CRISPR can modify traits related to stress responses in quail. For instance, by editing genes that regulate stress hormones, it may be possible to produce quail that are less susceptible to stressors in their environment, leading to improved welfare outcomes (Khatri et al. 2019; Lee et al. 2019). Furthermore, advancements in gene‐editing techniques can facilitate the development of quail with enhanced reproductive traits (Khwatenge and Nahashon 2021), which can help maintain population stability and genetic diversity, crucial for long‐term welfare. Therefore, as research and technology continue to evolve, CRISPR technology may likely play a crucial role in the advancement of the quail breeding program (Ahn et al. 2016), shaping of quail farming and improvement of quail welfare.

### 7.23 Genomic Selection

#### 7.23.1 Use of Genomic Data to Improve Welfare‐Related Traits and Case Studies

Genomic selection in quail is an emerging field that leverages genomic data to enhance various traits, particularly those related to welfare. The use of genomic information allows for more precise breeding strategies aimed at improving the health, behaviour and overall welfare of quail (Morris et al. 2020; Dalmau et al. 2024). For instance, studies have shown that the composition of the gut microbiota can significantly influence the efficiency traits in Japanese quail, suggesting that genomic selection could be utilised to select favourable microbiota profiles that enhance welfare and productivity (Hosoda et al. 2024).

Case studies in poultry have illustrated the practical applications of genomic selection. In one study, the implementation of genomic selection in commercial breeding programs resulted in significant improvements in growth rates and feed efficiency, which are critical for both economic viability and animal welfare. Additionally, the use of genomic data has facilitated the identification of genetic markers associated with welfare traits, enabling breeders to make informed decisions that align with welfare standards.

### 7.24 Integration of Genetics With Environmental and Nutritional Innovations

#### 7.24.1 Synergistic Effects of Combining Genetic Advances With Enrichment and Nutrition

The integration of genetics with environmental and nutritional innovations in quail farming presents a promising avenue for enhancing productivity and sustainability. The advancements in genetic engineering can be leveraged by farmers to select for desirable characteristics, such as rapid growth rate, hardiness, disease resistance and overall health from a pool of genetic breakthroughs to optimise production under various environmental conditions (Hassan and Fadhil 2019). For example, research has shown that specific lines of quail react differently to varied dietary composition, suggesting a genotype‐by‐environment interaction that can be exploited to improve performance under a given dietary plan (Hassan and Fadhil 2019).

The inclusion levels of nutrients, such as amino acids and vitamins, have been manipulated to match the genetic profile of quails. Synergistic results have been recorded for this genetic‐nutrition modification, and significant benefits have also been reported. Additionally, the role of the microbiome in quail nutrition cannot be overlooked. Genetic variations among quail can influence gut microbiota composition, which, in turn, affects nutrient absorption and overall health. By understanding these interactions, farmers can implement targeted nutritional strategies that not only support the genetic potential of their quail but also promote a healthier gut environment, leading to improved growth and resilience against diseases.

#### 7.24.2 Examples of Integrated Approaches Leading to Enhanced Welfare

The integration of multiple approaches to enhance animal welfare has recently gained increased attention. One example of this integrated approach is the adoption of integrated pest management (IPM) strategies in livestock systems. In IPM systems, biological, cultural and chemical practices are combined to eliminate pests without causing damage to the animals or degrading the environment (Ehler 2006; Falkenberg et al. 2022). This holistic approach protects animal health and promotes better living conditions by reducing the need for harmful pesticides. Moreover, incorporating good animal welfare practices into breeding programs is another integrated approach that has improved the welfare of quails (Muhammad and Mirza 2019; Neeteson et al. 2020). Typically, parent stocks are selected to favour the traits that enhance welfare. Such traits might include tolerance to stress, disease tolerance or temperament, all of which can contribute to the birthing of a generation better suited to the environment.

##### 7.24.2.1 Ethical Considerations in Genetic Manipulation

The genetics of quails have been improved over the years, and the stocks we see today are a result of several years of genetic engineering efforts. It is worth stating there are broad ethical considerations in the genetic manipulation of quails, and these encompass a range of complex issues that arise from the exploration of genetic engineering technologies to improve them. The possibility of unintended outcomes is one of such issues (Chen et al. 2020). Usually, in genetic manipulation, there is an off‐target effect where modification for a particular trait of interest inadvertently yields unintended consequences through the alteration of other genes (Chen et al. 2020). This might lead to unforeseen welfare productivity issues for the birds and health issues for the consumers and has raised questions about the long‐term safety issues of genetically modified organisms in both the agricultural context and in the field of medicine.

Genetic manipulation has also not been able to attain a common ground with animal welfare, with ethical debate constantly arising with regard to these two ideas (Thompson 2010). Animal welfare generally emphasises the sentience of the animals and moves against imposing suffering on animals (Thompson 2010). The large number of animals often required for research and development can exacerbate these welfare issues, prompting calls for more humane approaches to genetic engineering.

Another persistent ethical consideration in genetic manipulation is the potential for misuse and the possibility of creating significant moral and social dilemmas, such as in the context of human enhancement or eugenics. As such, it is crucial for policymakers, scientists and ethicists to engage in ongoing discussions to establish guidelines and regulations that ensure responsible use of genetic technologies while addressing these ethical concerns. Moreover, the ethical implications of genetic manipulation can also extend to societal considerations such as social equity and access. It has often been discussed that advancement in genetic engineering technology could exacerbate existing inequalities, raising the question of who and what class of people have access to these technologies (Rubeis and Steger 2018; Bohua et al. 2023). It is, however, still undetermined if this will give rise to a new form of genetic elitism.

## 8 Future Directions in Quail Welfare Research

As reviewed in the previous section, technological advancements have proven to be beneficial in making environmental enrichments available to quails, and it is even more encouraging to know of the enormous possibilities for future innovations in quail husbandry because it can both help to enhance the welfare of the birds as well as improve the efficiency of farming practices. There is growing optimism for the integration of smart farming technologies in quail production. Automated systems for monitoring environmental conditions, such as temperature, humidity and light, are becoming increasingly sophisticated. These systems can adjust conditions in real‐time to mimic natural habitats, promoting natural behaviours like foraging and social interaction, which are essential for quail welfare.

Another promising innovation is using advanced feeding systems that encourage foraging behaviour (Miller and Mench 2006). These systems can include movable feeders that change locations periodically, stimulating exploration and reducing welfare problems associated with confinement. Additionally, incorporating varied substrates for dust bathing and nesting fulfils their biological needs, enhancing their overall welfare (Scholz et al. 2010; Monckton et al. 2020). Interactive enrichments provided by sensory stimulations have also been explored by research for the advancement of animal welfare, with studies showing that visual and auditory stimuli can enhance the engagement of animals (Wells 2009; Whittaker et al. 2023). Moreover, feed and feeding of quails are also being explored to improve quail husbandry, and this has led to the integration of diverse food options, such as live insects and sprouted seeds, which not only provide nutritional benefits but also encourage natural foraging behaviours (Deori et al. 2022).

Future directions in quail welfare research are progressively emphasising the incorporation of emerging technologies to improve the overall well‐being of these birds within agricultural settings. A pivotal area of advancement is the implementation of precision farming techniques, which employ data analytics and sensor technologies to continuously monitor the health and behaviour of quail in real‐time (Rowe et al. 2019; Kooij and Rutter 2020; Olejnik et al. 2022). This methodology enables farmers to make informed decisions aimed at enhancing animal welfare by effectively addressing challenges such as stress, overcrowding and adverse environmental conditions.

**FIGURE 3:**
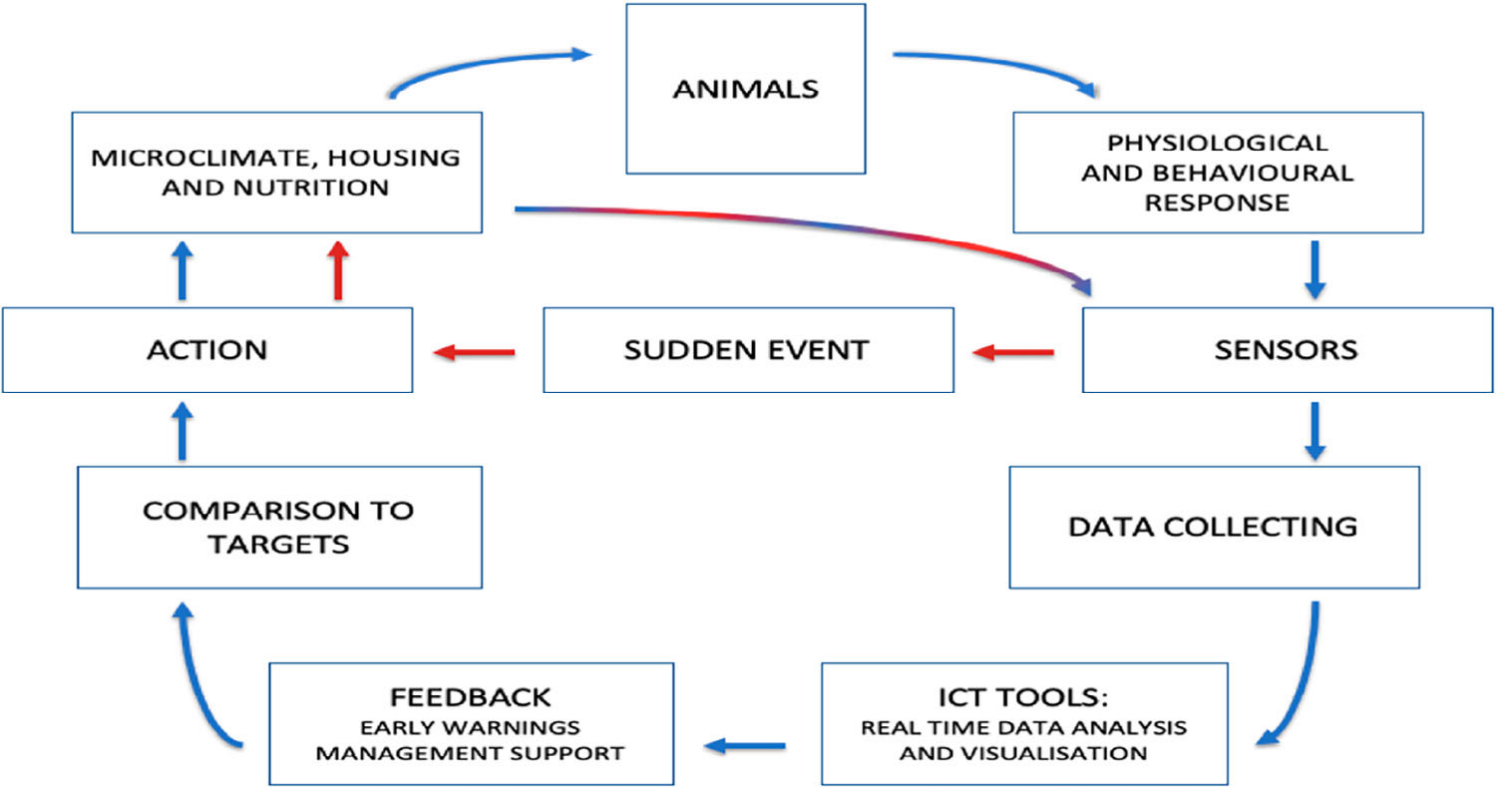
Schematic overview of farm management optimisation through the implementation of precision livestock farming tools. *Source*: Adapted from Olejnik et al. (2022).

The integration of precision farming and artificial intelligence (AI) automation is increasingly recognised as essential in the domain of welfare monitoring within modern agriculture, particularly concerning livestock management (Neethirajan 2024). The schematic overview of farm management optimisation through the implementation of precision livestock farming (PLF) tools is shown in Figure 3. The precision farming utilises advanced technologies, including AI, data analytics and sensor systems, to facilitate real‐time monitoring of animal health and behaviour (Olejnik et al. 2022). This technological amalgamation enables a proactive stance on animal welfare, allowing potential issues to be addressed before they escalate. AI‐driven automation substantially enhances welfare monitoring by providing continuous surveillance of animal conditions. For example, automated systems can observe vital signs, feeding patterns and social interactions among livestock, enabling the early identification of stressors or health problems (Aydin et al. 2010). The collection of real‐time data is crucial for making informed decisions that improve animals’ living conditions and overall welfare.

Furthermore, precision farming technologies can optimise environmental conditions within agricultural facilities. Automated climate control systems can adjust temperature, humidity and ventilation based on real‐time data, ensuring optimal conditions for animals (Jones et al. 2005). This not only alleviates stress but also promotes better health outcomes. Additionally, AI can analyse behavioural patterns to discern social dynamics within animal groups (Landgraf et al. 2021). The implementation of automated systems for feeding, watering and environmental control significantly reduces human error, ensuring optimal care for quail. These systems can be programmed to adjust based on real‐time data, thereby promoting better living conditions and reducing stress among the birds. Ongoing research is also investigating the impact of social interactions and environmental enrichment on quail welfare. Understanding how these factors influence behaviour and health could lead to improved housing designs that cater to quails’ natural instincts, ultimately enhancing their quality of life.

The potential for real‐time welfare assessment and intervention in livestock management is rapidly advancing, driven by innovations in technology such as AI, sensor systems and data analytics (Neethirajan 2024). These advancements enable continuous monitoring of animal health and behaviour, facilitating timely interventions that can significantly enhance animal welfare. Real‐time welfare assessment involves automated systems that collect data on various indicators of animal well‐being, such as vital signs, feeding patterns and social interactions (Neethirajan 2024). For instance, AI algorithms can analyse this data to detect anomalies that may signal stress or health issues, allowing farmers to address problems before they escalate. This proactive approach not only improves the living conditions of animals but also enhances overall productivity and efficiency in farming operations. Overall, the future of quail welfare research stands to benefit significantly from emerging technologies that improve farming practices while prioritising the health and well‐being of quail populations. By adopting these innovations, the poultry industry can ensure a more sustainable and humane approach to quail farming.

## 9 Interdisciplinary Approaches to Quail Welfare

Interdisciplinary approaches to quail welfare are paramount for improving the wellbeing of these birds while addressing the multifaceted challenges of livestock production. The integration of animal science, technology and ethics is critical in formulating effective welfare strategies. Animal science offers valuable insights into the biological and behavioural needs of quails, which can inform improved housing, feeding and management practices. For example, research has demonstrated that environmental enrichment can significantly enhance quail welfare by enabling them to express natural behaviours, thereby reducing stress and promoting overall health (Recoquillay et al. 2013).

Technology also plays a vital role in advancing quail welfare. Innovations such as automated monitoring systems provide real‐time tracking of quail health and behaviour, facilitating timely interventions when issues arise (Michie et al. 2020). Furthermore, advancements in genetic research can lead to the development of quail breeds that are more resilient to stressors, thus improving their welfare under various farming conditions (Canario et al. 2013).

Ethical considerations are equally significant in this interdisciplinary framework. Prioritising the ethical treatment of quails ensures that their welfare is not compromised for productivity. This involves engaging with stakeholders, including farmers, researchers and animal welfare organisations, to establish guidelines that reflect humane practices. Opportunities for collaborative research and innovation are abundant in this field. Multidisciplinary teams can collaborate to explore innovative welfare assessment methods, develop sustainable farming practices and create educational programs that raise awareness about quail welfare among farmers and consumers. By fostering collaboration across disciplines, we can enhance quail welfare while also addressing broader issues related to animal agriculture and sustainability.

A major difficulty is the availability of funding to provide farms with innovative PLF systems. These often require expensive expenses with no short‐term benefits, so larger farmers are more likely to afford them, worsening the technical gap between large and small‐ to medium‐sized poultry producers (McFadden et al. 2022). A system of financial funding indirectly supported by local governments and national programs encouraging PLF technology (Baylis et al. 2022; Hasler et al. 2022). Werkheiser (2018) also point out that farms that utilise PLF solutions may limit job options because such technology is highly automated. Consequently, employees with less experience and fewer skills may be hired, potentially affecting the quality of their work.

Additionally, the environmental impact of energy‐intensive monitoring systems must be considered. To address these issues, integrating renewable energy sources to power these technologies can help reduce their carbon footprint and promote environmental sustainability.

Collaborative research and innovation are crucial for overcoming these obstacles. The agricultural industry may produce flexible solutions that address a wide range of demands and environments by developing collaborations among researchers, technologists and farmers. This collaborative approach may result in the development of standards and best practices that reflect humane and sustainable farming methods, ensuring that quail welfare is prioritised alongside productivity.

Although developing technologies can dramatically assist the future of quail welfare research, the accompanying problems must be addressed to realise their full potential. The use of technology in quail farming can be both humane and sustainable if robust data security measures are implemented, cost‐effective solutions are developed, and collaborative efforts are encouraged.

## 10 Conclusion

This review emphasises the revolutionary potential of combining environmental enrichment, optimal nutrition and genetic innovations to improve the health, productivity and general welfare of quails. Further research and advancements in quail welfare practices will continue to improve the well‐being of these birds and contribute to the sustainability of the quail industry. Although customised nutrition, such as functional feed additives and precision feeding, supports immunological responses and physiological resilience, techniques, like offering perches, dust‐bathing materials and light modulation, create more naturalistic habitats, lowering stress and encouraging positive behaviours. Furthermore, welfare‐friendly features can be selected without compromising production. These approaches represent a holistic pathway to sustainable quail production, aligning with societal and ethical expectations while ensuring economic viability. Prioritising welfare through science‐driven innovations will enhance production performance and set a benchmark for sustainability, animal care and profitability in quail production.

## Author Contributions

All authors contributed to the  conception and design of the study. Material preparation, data collection and analysis were performed by O. E. Oke, K. M. Oliyide, O. A. Akosile, A. I. Oni, E. O. Adekunle, B. O. Oyebanji, O. P. Aremu, I. M. Adeoba, T. A. Eletu and J. O. Daramola and all authors commented on previous versions of the manuscript. All authors read and approved the final manuscript.

## Ethics Statement

The authors have nothing to report.

## Conflicts of Interest

The authors declare no conflicts of interest.

## Peer Review

The peer review history for this article is available at https://publons.com/publon/10.1002/vms3.70424.

## Animal Welfare Statement

The authors confirm that the ethical policies of the journal, as noted on the journal's author guidelines page, have been adhered to.

## Data Availability

The data that support the findings of this study are available from the corresponding author upon reasonable request.
